# The Kinocidin Interleukin-26 Shows Immediate Antimicrobial Effects Even to Multi-resistant Isolates

**DOI:** 10.3389/fmicb.2021.757215

**Published:** 2021-10-18

**Authors:** Bjoern-Thore Hansen, Gregor Maschkowitz, Rainer Podschun, Helmut Fickenscher

**Affiliations:** Institute for Infection Medicine, Christian-Albrecht University of Kiel and University Hospital Schleswig-Holstein, Kiel, Germany

**Keywords:** kinocidin, interleukin-26, antimicrobial peptide, bactericidal activity, multi-resistant bacteria

## Abstract

The cationic proinflammatory cytokine Interleukin 26 (IL-26) shows antibacterial activity and inhibits the replication of cytomegalovirus and hepatitis C virus. This study evaluates the early microbicidal activities of IL-26 against major bacterial species including multi-resistant variants and *Candida albicans*. Recombinant IL-26 was bacterially expressed and studied for its microbicidal effects in culture. We show that IL-26 has strong 90% bactericidal activities against *Enterococcus faecalis, Enterococcus faecium, Staphylococcus aureus*, and *Acinetobacter baumannii*. Similarly, IL-26 sensitivity was also detectable in vancomycin-resistant Enterococcus species, methicillin-resistant *S. aureus*, and carbapenem-resistant *A. baumannii* clinical isolates. Additionally, a significant, albeit weak fungicidal effect against *Candida albicans* was observed. Activities against *Escherichia coli, Klebsiella pneumoniae*, and *Pseudomonas aeruginosa* were not detectable. The proinflammatory cytokine and kinocidin IL-26 shows strong bactericidal activities against *A. baumannii* and, almost selectively, against Gram-positive bacteria.

## Introduction

The highly cationic proinflammatory cytokine Interleukin 26 (IL-26) is a member of the IL-10 family of cytokines. IL-26 is produced by different cell types, such as activated T cells or inflammatory fibroblasts, and stimulates the production of other proinflammatory cytokines in various cell types (Knappe et al., 2000; Fickenscher et al., 2002; Hör et al., 2004; Corvaisier et al., 2012; Braum et al., 2013; Che et al., 2014, 2017; Larochette et al., 2019). IL-26 uses a specific, heterodimeric cytokine receptor consisting of IL-20R1 and IL-10R2 on epithelial cells, whereas its action on other cell types is independent of this receptor (Hör et al., 2004; Sheikh et al., 2004; Corvaisier et al., 2012). Accordingly, elevated IL-26 levels were found in inflamed tissues or plasma of patients with different inflammatory diseases (Dambacher et al., 2009; Corvaisier et al., 2012; Che et al., 2014, 2017; Miot et al., 2014; Meller et al., 2015; Konradsen et al., 2016; Fujii et al., 2017; Heftdal et al., 2017; Poli et al., 2017; Caiazzo et al., 2018; Larochette et al., 2019; Scala et al., 2019; Brilland et al., 2021). Moreover, IL-26 inhibits cytomegalovirus and hepatitis C virus replication and promotes the replication of vesicular stomatitis virus (Braum et al., 2013; Miot et al., 2014). IL-26 is a highly cationic protein with amphipathic helices, which are typical for cationic cell-penetrating peptides (Knappe et al., 2000; Meller et al., 2015). IL-26 shares structural similarity with antimicrobial peptides (AMP) and bacteriostatic activities of IL-26 against both Gram-positive and Gram-negative bacteria have been demonstrated (Meller et al., 2015; Agak et al., 2018; Woetmann et al., 2018; Scala et al., 2019). Recombinant IL-26 has a molecular weight of 19kDa and the formation of dimers, oligomers, and multimers has been described. Since IL-26 combines cytokine functions with antimicrobial activities, it belongs to the group of kinocidins (Yount et al., 2004; Yeaman et al., 2007; Larochette et al., 2019). This project targets the rapid, microbicidal activities of IL-26.

## Materials and Methods

### Production of Recombinant Protein

Induction of IL-26 protein synthesis was achieved in *Escherichia coli* strain XL1blue, which is capable of producing IL-26 in inclusion bodies, by adding 1mM isopropyl β-D-1-thiogalactopyranoside and cultivation for 6h (Knappe et al., 2000; Hör et al., 2004). First, the bacteria were disrupted mechanically in 6 M guanidinium chloride with 0.1M NaH_2_PO_4_, 0.01M Tris, and 100mM β-mercaptoethanol. Bacterial debris was removed by centrifugation for 30min at 8,873×g and by sterile filtration. Nickel-chelate affinity chromatography was used for protein purification under denaturing conditions (Ni Sepharose 6 Fast Flow, GE Healthcare Life Sciences, Buckinghamshire, UK). Particle-bound IL-26 was eluted under increasing imidazole concentrations (40mM, 500mM, and 600mM). Eluate fractions were tested for 19kDa bands in Coomassie-stained protein gels. Renaturation of relevant eluate fractions was achieved by dialysis for 24h twice, at 4°C (20mM HEPES, 1mM MgCl_2_, 20mM KCl, 0.1mM EDTA, 1% glycerol, 1mM oxidated glutathione, and 5mM reduced glutathione, pH 8.0).

### Testing IL-26 for Functionality

**Figure 1:**
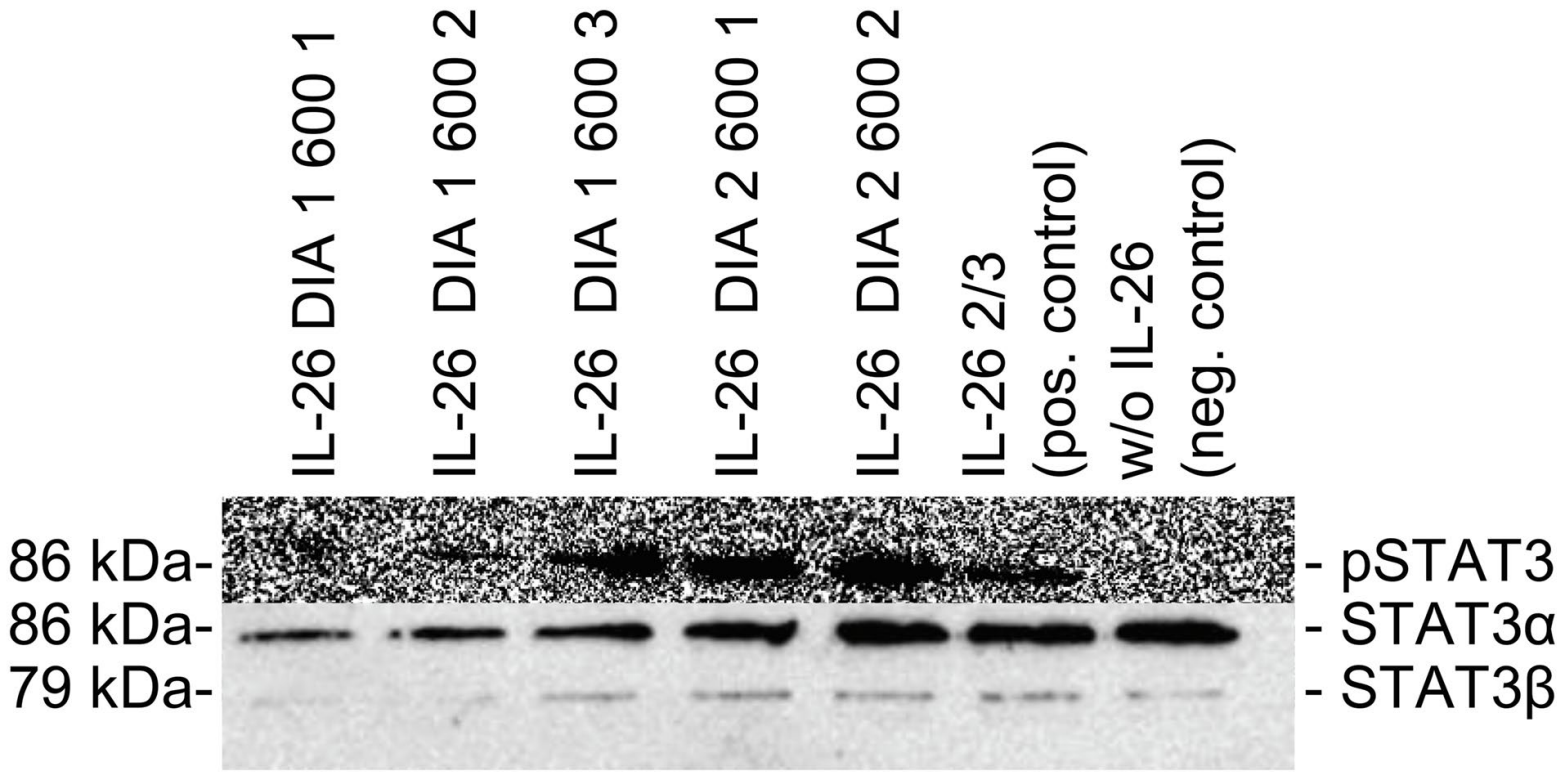
Western blot analyses of COLO-205 cell lysates treated with different batches of IL-26 dialysates, positive control, and negative control. **Top:** Detection of phospho-STAT3 using primary polyclonal rabbit-raised phospho-STAT3 antibodies. **Bottom:** Detection of STAT3α and STAT3β using primary polyclonal rabbit-raised STAT3 antibodies. For both analyses, horseradish peroxidase-conjugated anti-rabbit IgG antibodies were used as second reagents. Chemiluminescence detection was performed with a charge-coupled device camera (LAS-3000, Fujifilm, Japan).

The physiological STAT3 phosphorylation activity of the newly produced IL-26 was tested with the human colon carcinoma cell line COLO-205. IL-26 (5μg/ml) was added for 45min at 37°C. Cell lysates were separated on protein gels and Western blots were probed with anti-phospho-STAT3 and anti-STAT3 antibodies (anti-phospho-Tyr705 STAT3, #9131, rabbit; anti-STAT3, #9132, rabbit) and horseradish peroxidase-conjugated anti-rabbit IgG antibodies (#7074, goat, Cell Signaling Technology, Beverly, United States) as secondary reagent. Signals were detected by chemiluminescence (Figure 1) using a charged-coupled device camera (LAS-3000, Fujifilm, Tokio, Japan). If phospho-STAT3 bands were detectable, the respective IL-26 fraction was considered biologically active.

### Testing for Antimicrobial Activity

Bacteria and *C. albicans* were initially grown on Columbia sheep blood agar at 37°C overnight and then for 20h at 37°C in 10ml tryptic soy broth (TSB) medium with 10mM NaCl. Then, 50μl of the culture was added to 10ml of TSB/10mM NaCl medium and incubated for 3h at 37°C for reaching the logarithmic phase of growth and the optical density at 600nm was determined. We diluted the culture with 10mM NaCl to reach target concentrations of 10^5^CFU/ml. Next, 100μl of the microbial suspension was added to 100μl of different concentrated IL-26 solutions (100, 30, 10, 3, and 1μg/ml) to achieve final IL-26 concentrations in the wells of 50, 15, 5, 1.5, and 0.5μg/ml. We always ran negative controls with pure dialysis buffer instead of the IL-26 solution, as well as positive controls with bacteria that were previously shown to be highly sensitive to IL-26, when species with low or lacking IL-26 sensitivity were examined.

Immediately after the start of the treatment, and after 1, 2, 3, and 4h, 20μl from each culture was sampled and diluted in a 0.85% NaCl solution and 100μl of that dilution was pipetted on two lysogeny broth (LB) agar plates each. The plates were incubated overnight at 37°C and the colonies were counted. The numbers of colony forming units per milliliter (CFU/ml) were calculated after averaging of the counting results of the two separate plates in consideration of the dilution factor. Totally, 15,000 LB agar plates were plated by hand for 66 independent experiments.

### Statistical Evaluation

Statistical evaluation was performed with GraphPad PRISM 8 (GraphPad Software, Inc., San Diego, CA, United States). Values of *p* were determined by using the independent two-sample *t*-test. We used linear regression for calculating the minimal concentration for 90% bactericidal or fungicidal effects (MBC_90_/MFC_90_), respectively, the lethal dose or concentration for 90% killing (LD_90_/LC_90_). All results of this study are given as MBC_90_/MFC_90_, even though the use of LD_90_/LC_90_ is also common in AMP research. The use of the terms MBC/MFC may be more precise from a microbiological point of view. Results with values of *p* ≤0.05 were rated significant. As far as not given in numbers, significances are labeled as followed: ^*^p≤0.05; ^**^p≤0.01; ^***^p≤0.001; and ^****^p≤0.0001, ns=not significant.

## Results

IL-26 was tested for bactericidal activities against different Gram-positive and Gram-negative strains of different antibiotic resistance phenotypes, as well as for fungicidal activities against *C. albicans*. All experiments were performed with a standardized protocol and were highly reproducible. The anti-infective activities were analyzed quantitatively for the minimal concentration for 90% bactericidal or fungicidal effects (MBC_90_/MFC_90_).

### Activity Against Enterococci

**Figure 2:**
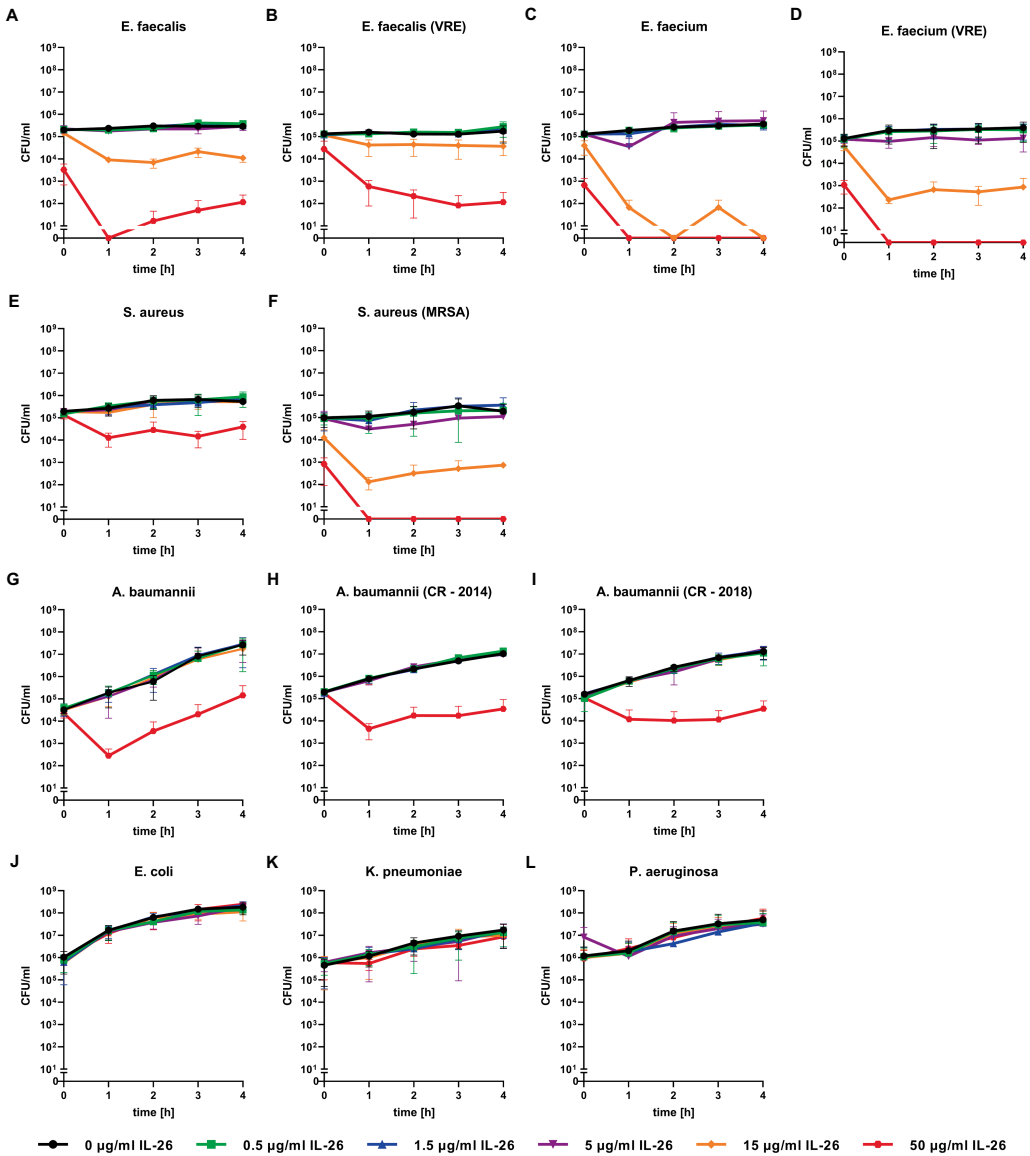
Number of colony forming units per ml of different bacteria over 4h incubation at different IL-26 concentrations. Panels **(A-F)** show IL-26 sensitive Gram-positive strains, panels **(G-I)** show IL-26 sensitive Gram-negative strains, and panels **(J-L)** show IL-26-resistant Gram-negative strains. Data are given as mean with standard deviation, for each strain *n*=3. CFU, colony-forming units; VRE, vancomycin-resistant Enterococcus; MRSA, methicillin-resistant *S. aureus*; and CR, carbapenem resistant.

Directly after adding 50μg/ml IL-26 to *Enterococcus faecalis* ATCC 29212 cultures, an immediate and highly significant (*p*≤0.0001) reduction of the number of colony forming units (CFU) of approximately 98.4% was detected. After 1h, significant effects were found from 5μg/ml on. Complete eradication of all bacteria was reached at 50μg/ml IL-26, after incubation for at least 1h. The CFU numbers after 2, 3, and 4h of incubation were reduced by 97.3, 92.3, and 96.1% at 15μg ml IL-26 (Figure 2A).

Similar to the antibiotic-sensitive type strain, a vancomycin-resistant clinical isolate of *E. faecalis* showed an instant significant effect. After 1h, significant CFU number reductions were found starting from 0.5μg/ml, whereas the strongest results were observed at 15μg/ml (reduction of 72.5%) and 50μg/ml (99.6%). The reduction levels stayed constant over time. After 4h, the incubation with 50μg/ml IL-26 resulted in a highly significant decline of CFU numbers by 99.95% (Figure 2B).

Likewise, *Enterococcus faecium* ATCC 6057 showed a prompt significant drop of the CFU numbers at 15μg/ml by 70.6% and at 50μg/ml by 99.5%. This effect increased, when the incubation was performed for 1h. Reduction values were 81.3% for 5μg/ml, 99.96% for 15μg/ml, and a complete killing of all bacteria was achieved for 50μg/ml. After 2 and 4h, complete eradications were reached at 15μg/ml. At 3h, reductions amounted to 99.98% for 15μg/ml and 100% for 50μg/ml (Figure 2C).

**Figure 3:**
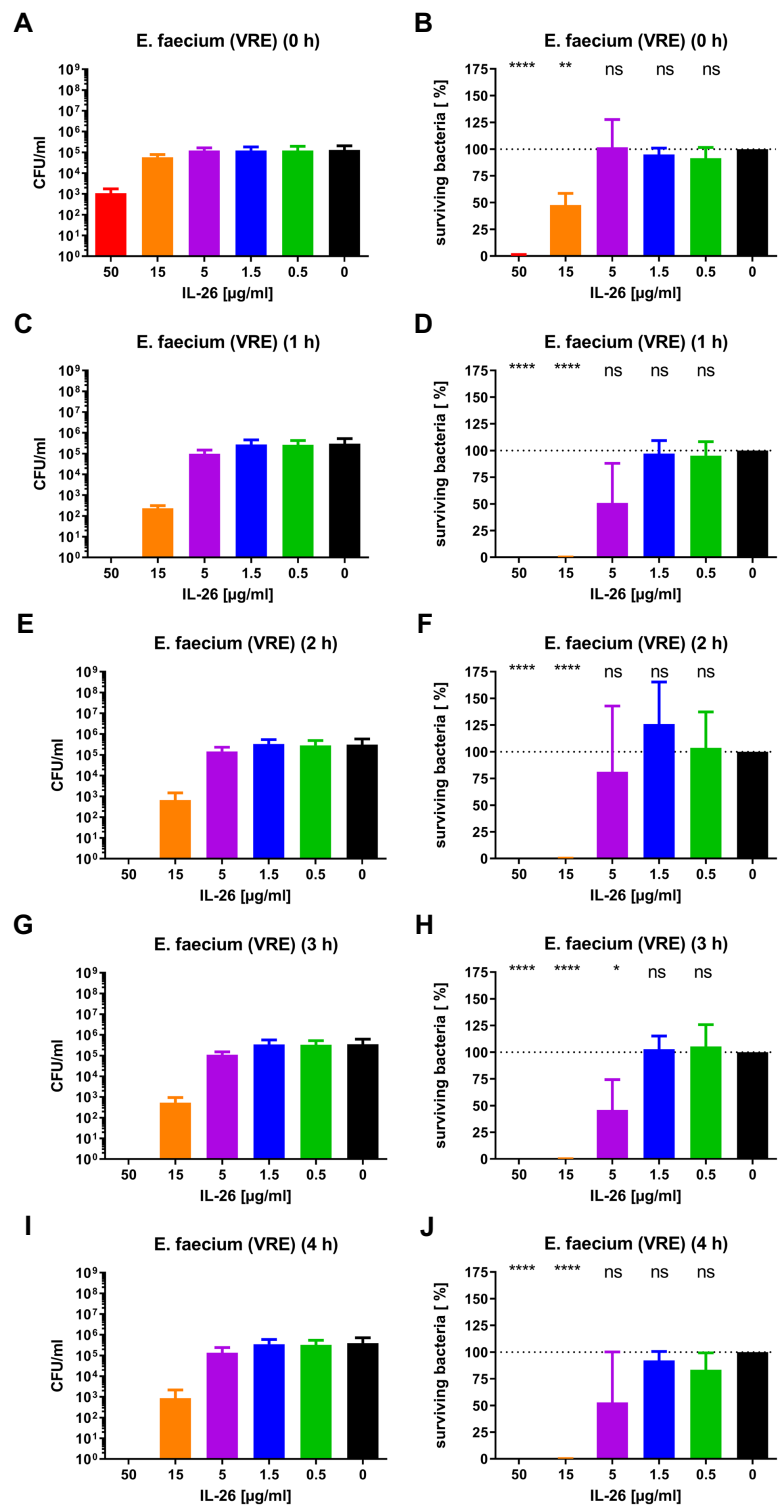
Number of colony forming units per ml **(A,C,E,G,I)** and percentage of surviving bacteria **(B,D,F,H,J)** for vancomycin-resistant *E. faecium* DSM 17050 for different incubation durations and different IL-26 concentrations. ^*^p≤0.05; ^**^p≤0.01; ^****^p≤0.0001; ns=not significant; and independent two-sample *t*-test. Mean with standard deviation, *n*=3. The colors refer also to Figure 2.

Subsequently, we checked for IL-26 sensitivity of vancomycin-resistant *E. faecium* DSM 17050 (Deutsche Sammlung von Mikroorganismen, German Collection of Microorganisms, Hannover, Germany). Again, a significant immediate decline by 99.1% was achieved at 50μg/ml IL-26. After 1h, complete eradication of all bacteria was reached at 50μg/ml, whereas 99.89% were killed at 15μg/ml. These effects were stable (± 0.2% at 15μg/ml) for 2, 3, and 4h incubation time. After 3h, an additional significant but weaker effect was found at 5μg/ml, which lost its significance after 4h (Figures 2D, 3).

### Activity Against *Staphylococcus aureus*

In the case of *S. aureus* ATCC 6538, we found an instant reduction of the CFU numbers of approximately 23.2% at 50μg/ml IL-26 (*p*=0.37), which increased after 1h to a highly significant reduction by 94.6% (*p*<0.0001). Further increments were achieved upon ongoing incubation. CFU reductions at 50μg/ml reached 95.6, 98.0, and 93.4% after 2, 3, and 4h. After 2 and 3h, significant but weak effects were detectable at 15μg/ml (Figure 2E).

In contrast to the antibiotic-sensitive *S. aureus* ATCC 6538, the MRSA strain ATCC 33593 showed highly significant CFU reductions immediately after adding IL-26 to the cultures at 15μg/ml with a decline by 90.1% and at 50μg/ml by 99.3%. These effects increased after 1h with CFU reductions by 53.3% at 1.5μg/ml, 81.4% at 5μg/ml, 99.8% at 15μg/ml, and a complete eradication of all bacteria at 50μg/ml. After 2h, the effects at 5μg/ml and 15μg/ml were stable (±0.1%) and complete killing at 50μg/ml was still achieved. When incubating for 3h, reductions were found at 0.5μg/ml (33.5%), 1.5μg/ml (36.5%), 5μg/ml (83.9%), 15μg/ml (99.8%), and 50μg/ml (100%), of which all were significant, besides at 1.5μg/ml. Last, after 4h, declines by 63.6% at 5μg/ml, 98.6% at 15μg/ml, and complete eradication at 50μg/ml were observed (Figure 2F).

### Activity Against Multi-resistant Gram-positive Bacteria

The MBC_90_ for immediate effects ranged from 38.5μg/ml (MRSA ATCC 33593) to 57.5μg/ml (vancomycin-resistant *E. faecalis*), except for the methicillin-sensitive *S. aureus* ATCC 6538 with an unusually high MBC_90_ of 136μg/ml. Two groups of similar MBC_90_ ranges were defined. In the first group, *E. faecalis* ATCC 29212, *E. faecium* ATCC 6057, vancomycin-resistant *E. faecium* ATCC 17050, and MRSA ATCC 33593 had average MBC_90_ values between 12.5μg/ml and 13.4μg/ml IL-26 for 1 to 3h and 19.3μg/ml after 4h. In the other group, the average MBC_90_ for 1 to 4h for vancomycin-resistant *E. faecalis* and methicillin-sensitive *S. aureus* ATCC 6538 ranged between 43.7μg/ml and 45.5μg/ml (Table 1). Hence, we were able to show for the first time that IL-26 functions as a highly active bactericidal agent against different Gram-positive bacteria. These effects are independent of the antibiotic resistance phenotypes since multi-resistant strains of *S. aureus, E. faecalis*, and *E. faecium* were as sensitive as or even more sensitive than their antibiotic-sensitive counterparts.

**Table 1:** MBC_90_/MFC_90_ values for all IL-26-sensitive species.

MBC_90_/MFC_90_					
Species
	0	1	2	3	4	*h*
*E. faecalis*, ATCC 29212	45.58	14.16	14.23	14.40	14.51	μg/ml
*E. faecalis* (VRE)	54.48	40.04	41.59	40.67	42.38	μg/ml
*E. faecium*, ATCC 6057	46.75	11.72	13.58	13.79	13.41	μg/ml
*E. faecium* (VRE), DSM 17050	42.84	12.94	14.02	12.75	13.02	μg/ml
*S. aureus*, ATCC 6538	136.00	47.31	46.19	47.15	48.54	μg/ml
*S. aureus* (MRSA), ATCC 33593	38.48	11.51	11.87	11.42	36.20	μg/ml
*A. baumannii*, ATCC 11775	149.40	46.12	48.42	46.21	43.36	μg/ml
*A. baumannii* (CR), 2014	832.10	47.15	47.90	48.18	49.97	μg/ml
*A. baumannii* (CR), 2018	142.30	47.46	47.96	46.02	48.32	μg/ml
*C. albicans*, ATCC 24433	–	–	81.41	77.77	54.74	μg/ml

*Data calculated by linear regression; *n*=3 per species; “–”= not calculable*.

### Activity Against *Acinetobacter baumannii*

Concerning Gram-negative species, we first tested *A. baumannii* ATCC 19606. Similar to all tested Gram-positive bacteria, an initial albeit weak CFU reduction was observed. After 1h of incubation, a highly significant decline of 99.8% was detected at 50μg/ml IL-26 and stayed constant for 4h (±0.3%). Additionally, we found a 50% non-significant reduction at 15μg/ml (Figure 2G).

Moreover, two highly resistant *A. baumannii* CR isolates were analyzed, which solely were sensitive for colistin. The first one was isolated in 2014 from a patient from a local outbreak at the University Hospital Schleswig-Holstein in Kiel, Germany. An immediate reduction was not observed but, again, highly significant CFU reductions were measureable at 1, 2, 3, and 4h (99.4, 99.3, 99.7, and 99.7%) at IL-26 concentrations of 50μg/ml (Figure 2H). The other *A. baumannii* CR isolate resulted from a patient from 2018 with previous hospitalization in a country with high prevalence of colonization with CR *A. baumannii*. Here, we detected a significant immediate reduction of 31.7% at 50μg/ml IL-26. Furthermore, weaker and non-significant reductions were detectable at 0.5μg/ml, 5μg/ml, and 15μg/ml. After 1h, the decline of the CFU count at 50μg/ml IL-26 was 98.7% and increased in the course of the experiments to 99.4% (2h), 99.8% (3h), and 99.6% (4h; Figure 2I). The mean MBC_90_ values for the three *A. baumannii* strains (ATCC 19606, 2014, 2018) were 374.6μg/ml for 0h, 46.91μg/ml for 1h, 48.09μg/ml for 2h, 46.8μg/ml for 3h, and 47.22μg/ml for 4h (Table 1).

*A. baumannii* was the only tested Gram-negative species with IL-26 sensitivity. In contrast, we were unable to detect any antimicrobial effects against *E. coli* ATCC 11775, *K. pneumoniae* ATCC 4352, or *P. aeruginosa* ATCC 27853 for IL-26 concentrations up to 50μg/ml (Figures 2J–L).

### Activity Against *Candida albicans*

Last, the sensitivity of *C. albicans* ATCC 24433 against IL-26 was investigated. Immediate effects were not detected at up to 50μg/ml IL-26. The CFU reduction values at 50μg/ml IL-26 were 29% after 1h (non-significant), 67.5% after 2h, 58.2% after 3h, and 78.5% after 4h (all significant). The MFC_90_ values ranged from 54.7 to 81.41μg/ml (Table 1). Hence, we were able to show a reproducible and significant, albeit weak fungicidal activity of IL-26 against *C. albicans*.

Thus, the cytokine IL-26 with its proinflammatory, bactericidal, antiviral, and fungicidal activities can be attributed to the group of kinocidins which was defined for cytokines with direct antimicrobial effects, such as human mammalian platelet factor 4 (hPF-4; Yount et al., 2004; Yeaman et al., 2007; Larochette et al., 2019).

## Discussion

Whereas all tested Gram-positive bacterial strains were highly sensitive to IL-26, we observed a remarkable difference between *A. baumannii* strains and all other Gram-negatives (enterobacteria and *P. aeruginosa*). Thus, the questions arise why all other Gram-negative species are non-sensitive for IL-26 and what the essential factor is for the sensitivity of *A. baumannii*. The lipopolysaccharides (LPS) and especially the O-antigen, which is the outer chain of the LPS, are two known factors for AMP resistance in Gram-negative bacteria (Silhavy et al., 2010; Joo et al., 2016). Acinetobacter species are unable to produce complete LPS due to the absence of O-antigen-ligase activity and the lipooligosaccharide (LOS) core, the lipid A, is remaining (Weber et al., 2016). Thus, O-antigen might be responsible for the IL-26 resistance of Gram-negative species.

Concerning the mode of IL-26 action, the direct interaction with the bacterial cell membrane including pore formation seems likely, similarly to other AMP (Patel and Akhtar, 2017). Due to its high cationic charge, IL-26 binds to glycosaminoglycans of the surface of eukaryotic cells (Hör et al., 2004), as well as to LPS and lipoteichoic acid of the surface of bacteria (Meller et al., 2015). Based on electron micrographs of *P. aeruginosa* ATCC 27853, bleb-formation followed by membrane disruption was described as the mode of IL-26 action (Meller et al., 2015). However, this needs to be interpreted with caution, since exactly the same *P. aeruginosa* strain has been classified as IL-26 resistant in this study.

The initial publication concerning antimicrobial activities of IL-26 described bacteriostatic effects at 50% level for 5 to 10μM IL-26 against *P. aeruginosa* ATCC 27853, *E. coli* ATCC 11775, *K. pneumoniae* O1:K2, and *S. aureus* ATCC 6538 but no detectable effects against *E. faecalis* ATCC 29212 and *C. albicans* ATCC 24433 (Meller et al., 2015). In contrast, our study was able to demonstrate strong bactericidal activities at 90% level against the Gram-positive strains *E. faecalis* ATCC 29212, vancomycin-resistant *E. faecalis, E. faecium* ATCC 6057, vancomycin-resistant *E. faecium* DSM 17050, *S. aureus* ATCC 6538, and MRSA ATCC 33593. Furthermore, we were able to show strong effects on naturally O-antigen deficient *A. baumannii* independently of carbapenem resistance, and weak effects on *C. albicans* ATCC 24433 after at least 2h of incubation. Effects against *E. coli* ATCC 11775, *K. pneumoniae* ATCC 4352, and *P. aeruginosa* ATCC 27853 were not detectable. This goes in line with a study in which IL-26 exhibited bacteriostatic activities against *S. aureus* ATCC 6538 but not against *P. aeruginosa* ATCC 27853 (Scala et al., 2019). Another published study did not show immediate effects for *S. aureus* and *E. coli*; however, bactericidal effects were detected after 4 to 24h of incubation with 1μg/ml IL-26 (Agak et al., 2018). In a third publication, an activity against biofilm formation of *S. aureus* was detected which was more pronounced for IL-26 than for the AMP LL-37 (Woetmann et al., 2018). Regarding Mycobacteria (M.) IL-26 has been shown to inhibit growth and reduce viability of *M. leprae* and *M. tuberculosis* in axenic cultures as well as within macrophages, probably by inducing lysis by bleb-formation after interaction with lipoarabinomannan (Dang et al., 2019; Hawerkamp et al., 2020).

The reason for these functional differences might be due to the different sources and qualities of commercially available IL-26 (Knappe et al., 2000; Hör et al., 2004). In order to ensure high quality and functionality, we used self-produced IL-26 which was functionally tested for STAT3 activation in a colonic carcinoma cell line. Moreover, molar concentrations are difficult to interpret since IL-26 occurs as monomers, dimers, oligomers, and even multimers (Knappe et al., 2000; Fickenscher et al., 2002; Meller et al., 2015).

Concerning the raising relevance of antibiotic-resistant bacteria, AMP have come under scrutiny, especially due to the lack of resistance development, even though they have always been present during evolution (Zasloff, 2002; Gallo and Hooper, 2012). Besides potential pharmacotechnological and galenic challenges in the production of this instable kinocidin, additional immunologic effects of the proinflammatory IL-26 need to be considered in the case of a possible therapeutic application. In published experiments, IL-26 or LL-37 was applied nasally in mice after nasal application of bacteria and a certain reduction of the CFU number (factor 10–100) was seen (Meller et al., 2015). However, these experiments were performed with *K. pneumoniae*, which was classified as IL-26 resistant in this study.

Compared to other AMP, which usually have a broad activity spectrum against Gram-positive and Gram-negative bacteria, protozoa, and fungi (Ebbensgaard et al., 2015), IL-26 has its main target in Gram-positive bacteria, which has not yet been described for other AMP. Thus, IL-26 is a unique member of the family of cationic AMP. In summary, we showed for the first time that IL-26 is a proinflammatory kinocidin with bactericidal and fungicidal activities, which is also active against *A. baumannii* and *C. albicans* and kills Gram-positive bacteria almost selectively.

## Data Availability Statement

The original contributions presented in the study are included in the article, further inquiries can be directed to the corresponding author.

## Author Contributions

BTH wrote the manuscript, designed and performed the experiments, and prepared the figures and tables. GM contributed to the experiments, reviewed and edited the manuscript, and supervised the project. RP gave advice for the experimental design and reviewed and edited the manuscript. HF wrote the manuscript, gave advice for the experimental design, and supervised the project. All authors contributed to the article and approved the submitted version.

## Funding

This project was supported in part by the Excellence Clusters Inflammation at Interfaces and Precision Medicine at Kiel and funded in part by the Deutsche Forschungsgemeinschaft (DFG, German Research Foundation) – project number 413490537 within the Kiel Clinician Scientist Programme in Evolutionary Medicine. We acknowledge financial support by the state Schleswig-Holstein within the funding programme Open Access Publikationsfonds.

## Conflict of Interest

The authors declare that the research was conducted in the absence of any commercial or financial relationships that could be construed as a potential conflict of interest.

## Publisher’s Note

All claims expressed in this article are solely those of the authors and do not necessarily represent those of their affiliated organizations, or those of the publisher, the editors and the reviewers. Any product that may be evaluated in this article, or claim that may be made by its manufacturer, is not guaranteed or endorsed by the publisher.
